# A novel strategy for interpreting the T-SPOT.*TB* test results read by an ELISPOT plate imager

**DOI:** 10.1371/journal.pone.0222920

**Published:** 2019-09-25

**Authors:** Tae Yeul Kim, Ho Eun Chang, Seong-Wook Lee, Soo Hyun Seo, Yun Ji Hong, Jeong Su Park, Kyoung Un Park

**Affiliations:** 1 Department of Laboratory Medicine, Seoul National University Bundang Hospital, Seongnam-si, Gyeonggi-do, Korea; 2 Department of Molecular Biology, Dankook University, Yongin-si, Gyeonggi-do, Korea; 3 Department of Laboratory Medicine, Seoul National University College of Medicine, Seoul, Korea; The University of Georgia, UNITED STATES

## Abstract

**Background:**

The T-SPOT.*TB* can be read by an ELISPOT plate imager as an alternative to a labor-intensive and time-consuming manual reading, but its accuracy has not been sufficiently discussed to date.

**Methods:**

1,423 test results obtained from manual reading using a microscope and an ELISPOT plate imager were compared. The agreement of qualitative test results was assessed using Cohen's kappa coefficient. The relationship of spot counts was studied using Bland-Altman analysis.

**Results:**

The overall percent agreement of the qualitative test results was 95.43% with a kappa coefficient of 0.91. Positive test results with the maximum net spot count of 8 and borderline test results showed relatively high discordance. The agreement of spot counts in panel A, panel B, and nil control was good, and variability did not increase with higher spot counts. On the basis of study findings, a novel strategy for interpreting the test results by an ELISPOT plate imager was proposed.

**Conclusions:**

To increase diagnostic accuracy, positive test results with the maximum net spot count of 8 and borderline test results should be manually confirmed. Our strategy could be a practical guide for laboratories to build their own strategies for interpreting the test results by an ELISPOT plate imager.

## Introduction

Tuberculosis (TB) remains a major public health threat worldwide, with 10.0 million new cases and 1.6 million deaths estimated to have occurred in 2017 [1]. One of the most daunting challenges for global TB control is to contain the reservoir of latent TB infection (LTBI), affecting an estimated 1.7 billion individuals, just shy of a quarter of the global population [2]. Without treatment, 5 to 10 percent of individuals with LTBI progress to active TB in their lifetime [3]. World Health Organization recommends that either a tuberculin skin test (TST) or interferon-gamma release assay (IGRA) can be used to test for LTBI [4]. IGRAs appear to be more specific than the TST for the diagnosis of LTBI, measuring interferon-gamma produced by T-cells in response to *Mycobacterium tuberculosis*-specific antigens absent from all Bacille Calmette-Guérin strains and most non-tuberculous mycobacteria [5].

The T-SPOT.*TB* (Oxford Immunotec, Abingdon, UK), the fourth IGRA to be approved by the U.S. Food and Drug Administration (FDA), uses an enzyme-linked immunospot (ELISPOT) method in which interferon-gamma released by antigen-specific T-cells is captured in the immediate vicinity of the T-cells by specific antibodies on the surface of a membrane and then visualized as spots. The number of spots can be counted by laboratory technicians using a hand held magnifying glass or a suitable microscope. However, such manual spot counting is a highly laborious and subjective process. Alternatively, automated ELISPOT plate imagers can be used to reduce the workload and subjectivity of manual spot counting. Each ELISPOT plate imager has a given set of reading parameters that define spots, which can be adjusted by the user’s preference [6]. Evaluating the accuracy of ELISPOT plate imagers is a very crucial task as inaccurate spot counting could potentially complicate clinical decision making for LTBI treatment. Few studies have addressed the variability of the T-SPOT.*TB* test results read by technicians and ELISPOT plate imagers [7, 8]. No studies, however, have investigated how to interpret the T-SPOT.*TB* test results by an ELISPOT plate imager.

In this study, the T-SPOT.*TB* test results read by laboratory technicians using a microscope and by an ELISPOT plate imager were compared. On the basis of study findings, we recommended a novel strategy for interpreting the T-SPOT.*TB* test results by an ELISPOT plate imager.

## Materials and methods

### T-SPOT.*TB*

The T-SPOT.*TB* was performed according to the manufacturer’s instructions. The assay requires four wells with different stimulations to be used for each patient sample: one as an antigen-free nil control, one as a positive control containing phytohemagglutinin, and two as panels A and B containing *M*. *tuberculosis*-specific antigens early secretory antigenic target-6 and culture filtrate protein-10, respectively. Spot counting was performed by one technician using a Nikon Eclipse E600 microscope (Nikon Corp., Tokyo, Japan) and then automatically on an ELISPOT plate imager (AID ELISPOT reader classic; Autoimmun Diagnostika GmbH, Strassberg, Germany) with software version 7.0, build 14733. A total of four technicians conducted manual spot counting during the study period. Reading parameters of the ELISPOT plate imager preset by the manufacturer were not adjusted throughout the study. Technicians did not quantify spot counts above 20 in a given sample well, annotating the well as ‘too many to count (TMTC)’. Positive controls giving less than 20 spot counts on the ELISPOT plate imager were sent for manual review and annotated as ‘TMTC’ when the spots were confluent. Test results were qualitative and reported as positive, negative, borderline, or invalid depending on the number of spots counted. The test result was interpreted as positive when the spot count in net panel A (panel A minus nil control) and/or net panel B (panel B minus nil control) was greater than or equal to 8. The test result was interpreted as negative when the spot count in both net panel A and net panel B was less than or equal to 4. A borderline test result was determined when the higher of the net panel A and net panel B spot count was 5, 6, or 7. The test result was classified as invalid when the spot count in the positive control was less than 20 and the spot count in net panel A and net panel B was less than or equal to 4. An invalid test result was also determined when the spot count in the nil control was greater than or equal to 10.

### Statistical analyses

Statistical analyses were performed using MedCalc for Windows, version 18.11.3 (MedCalc Software, Ostend, Belgium). The agreement of qualitative test results by the technicians’ manual reading and ELISPOT plate imager was assessed using Cohen's kappa coefficient (0.81 < κ < 1.00, very good; 0.61 < κ < 0.80, good; 0.41 < κ < 0.60, moderate; 0.21 < κ < 0.40, fair; κ < 0.2, poor). Cohen’s kappa coefficient was also calculated for each technician. The agreement and relationship of spot counts read by the technicians and ELISPOT plate imager were studied using Bland-Altman analysis. For samples with spot counts less than 20 in panel A, panel B, and nil control, the spot count differences between the ELISPOT plate imager and technicians’ manual reading were plotted against their means. The mean difference and its limits of agreement (±1.96 SD) were computed. The Institutional Review Board of Seoul National University Bundang Hospital approved the study protocol and waived the need for informed consent because no patients were at risk (IRB no. B-1907-550-102). Patient records were anonymized and de-identified prior to analysis.

## Results

**Fig 1:**
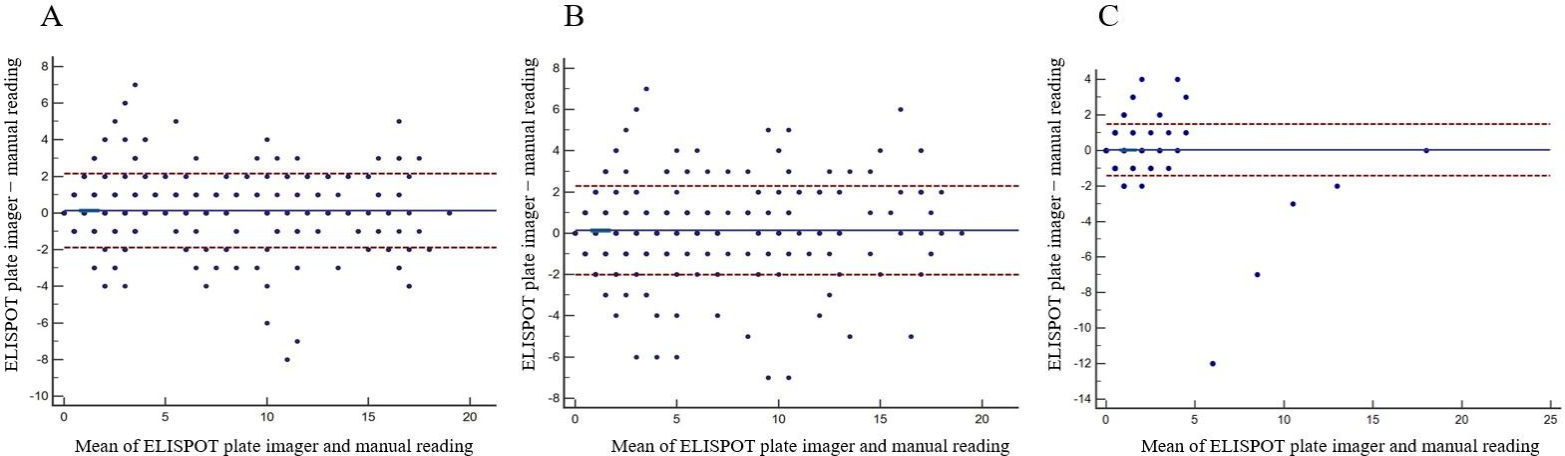
Bland–Altman plot of spot count differences between the technicians’ manual reading and ELISPOT plate imager. (A) Spot count differences in panel A; (B) spot count differences in panel B; (C) spot count differences in nil control. The horizontal solid lines (blue color) indicate the mean spot count difference, and the dashed lines (red color) represent the ±1.96 SD limits from the mean spot count difference.

Between January and November 2018, a total of 1,423 samples from 1,343 individuals were tested by the T-SPOT.*TB* at Seoul National University Bundang Hospital. Among 74 individuals retested at different time points, 68 individuals were tested twice, and 6 individuals were tested three times. The qualitative test results obtained from the technicians’ manual reading and ELISPOT plate imager were compared in Table 1. The qualitative test results read by each technician and ELISPOT plate imager were compared in S1 Table. The overall percent agreement of the qualitative test results was 95.43% (1358/1423) with a kappa coefficient of 0.91 (95% CI, 0.89 to 0.93), indicating very good agreement. Invalid test results showed the highest concordance (100%, 43/43), followed by negative and positive test results (99.00%, 893/902 and 97.82%, 359/367). Borderline test results showed the lowest concordance (56.76%, 63/111). Each technician’s kappa coefficient ranged from 0.87 to 0.93, indicating very good agreement (Table 2). The Bland-Altman analysis showed agreement of spot counts between the technicians’ manual reading and ELISPOT plate imager with a mean difference of 0.12 (limits of agreement: -1.91 to 2.15) for panel A, 0.13 (limits of agreement: -2.03 to 2.28) for panel B, and 0.01 (limits of agreement: -1.44 to 1.46) for nil control. Visual inspection of the Bland-Altman plot revealed that variability did not increase with higher spot counts (Fig 1).

**Table 1:** Comparison of the qualitative test results read by the technicians and ELISPOT plate imager.

	ELISPOT plate imager					
Technicians	Test results	Positive	Borderline	Negative	Invalid	Total
	Positive	359	8	0	0	367 (25.79%)
	Borderline	5	63	5	0	73 (5.13%)
	Negative	2	33	893	0	928 (65.21%)
	Invalid	1	7	4	43	55 (3.87%)
	Total	367 (25.79%)	111 (7.80%)	902 (63.39%)	43 (3.02%)	1423 (100.00%)

**Table 2:** Concordance and kappa coefficients of the qualitative test results read by the technicians and ELISPOT plate imager.

	Concordance % (no. of concordant results/total no. of results)					Kappa coefficient
	Overall	Positive	Borderline	Negative	Invalid	Kappa (95% CI)
Technicians, total	95.43% (1358/1423)	97.82% (359/367)	56.76% (63/111)	99.00% (893/902)	100% (43/43)	0.91 (0.89 to 0.93)
Technician 1	92.13% (234/254)	98.25% (56/57)	50.00% (13/26)	98.80% (164/166)	100% (5/5)	0.87 (0.81 to 0.93)
Technician 2	96.44% (379/393)	99.05% (104/105)	56.00% (14/25)	99.18% (241/243)	100% (20/20)	0.93 (0.90 to 0.97)
Technician 3	95.51% (660/691)	97.81% (179/183)	58.93% (33/56)	99.09% (434/438)	100% (14/14)	0.91 (0.88 to 0.94)
Technician 4	95.29% (81/85)	90.91% (20/22)	75.00% (3/4)	98.18% (54/55)	100% (4/4)	0.91 (0.83 to 0.99)

**Fig 2:**
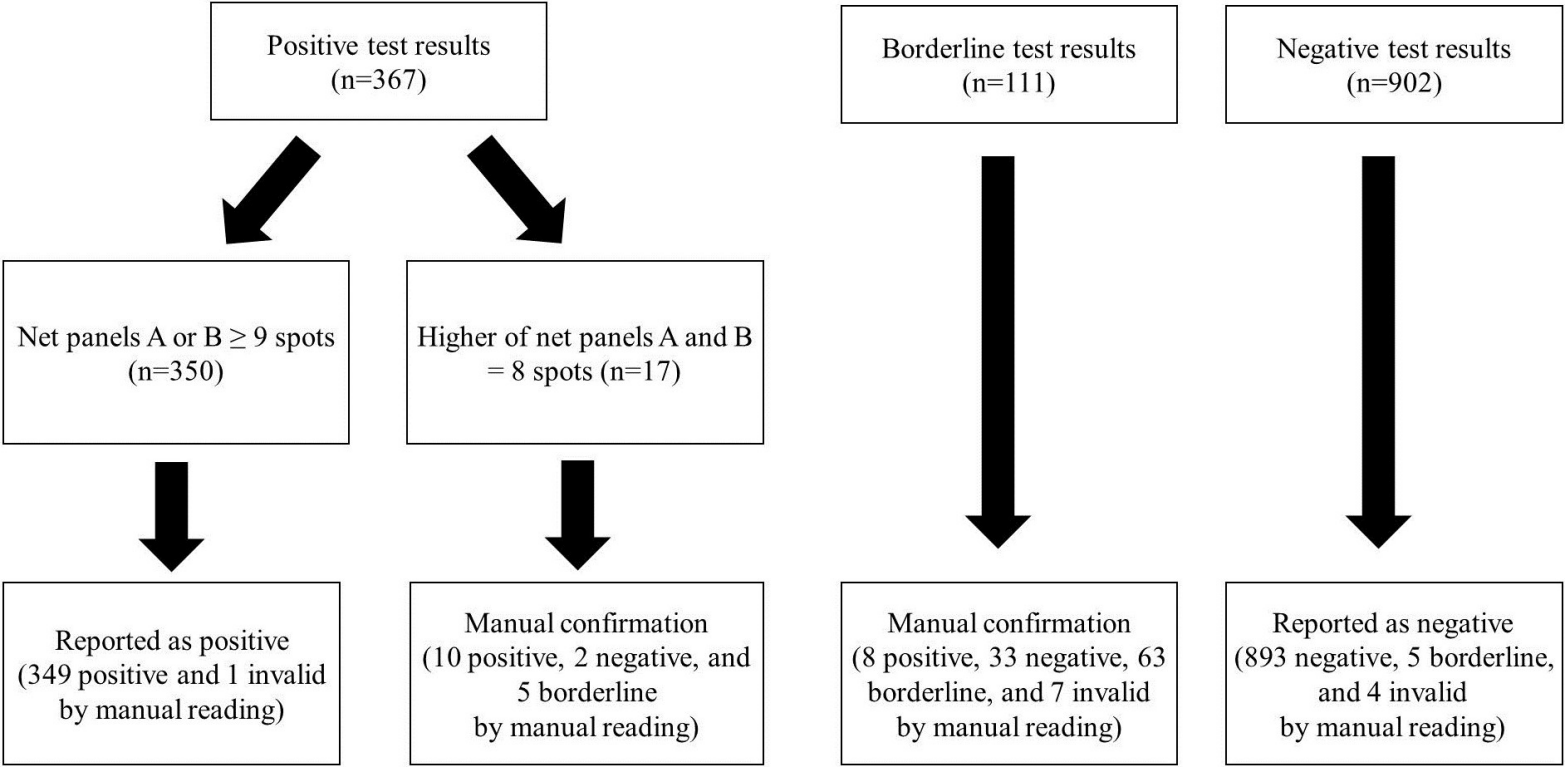
A novel strategy for interpreting the T-SPOT.*TB* test results by an ELISPOT plate imager on the basis of study findings. The test result is reported as positive if the spot count in net panel A or net panel B is greater than or equal to 9. Manual confirmation is warranted for borderline test results and positive test results with the maximum net spot count of 8. Negative test results are reported as such.

On the basis of study findings, we developed a new strategy for interpreting the T-SPOT.*TB* results read by an ELISPOT plate imager (Fig 2). Samples with the maximum net spot count greater than or equal to 9 are reported as positive because only one discordant test result (an invalid test result by manual reading) occurred in those samples. Samples with the maximum net spot count of 8 require manual confirmation due to the great variability (2 negative and 5 borderline test results by manual reading). Samples with borderline test results also warrant manual confirmation by virtue of the lowest concordance (8 positive, 33 negative, and 7 invalid test results by manual reading). Samples with negative test results are reported as negative, as only 9 out of 902 samples showed discordant test results (5 borderline and 4 invalid test results by manual reading). Retesting was conducted on 6 out of these 9 samples, producing 5 negative test results and one invalid test result. Invalid test results are reported as such because no discordant test results were observed in this study.

## Discussion

Accurate identification of individuals with LTBI is essential for clinical decision making. False positive results could result in unnecessary treatment, while false negative results would mean a missed opportunity to prevent active TB [9]. Therefore, variability of the T-SPOT.*TB* test results arising from different counting methods might have a huge economic impact. In the absence of a gold standard, agreement between different counting methods is a measure of diagnostic accuracy. This study aimed at investigating the diagnostic accuracy of an ELISPOT plate imager in comparison with manual reading using a microscope. The overall agreement of the qualitative test results read by the technicians and ELISPOT plate imager was very good with a kappa coefficient of 0.91, exceeding those reported in the previous studies [7, 8]. Additionally, the agreement of spot counts was found between the technicians and ELISPOT plate imager. The overall agreement of qualitative test results can be affected by the proportion of LTBI in the study population [7]. As positive and negative test results showed comparable concordance (97.82% and 99.00%, respectively), the overall agreement was unlikely to be affected by the prevalence of LTBI in the study population.

There was a very good agreement of the qualitative test results between each individual technician and the ELISPOT plate imager with kappa coefficients ranging from 0.87 to 0.93. Such consistent agreement might indicate that spot counting tendency of each individual technician does not significantly affect test results and clinical decision making. However, a limitation of this study was that we did not directly measure the inter-technician variability of the T-SPOT.*TB* test results. Although technicians are trained to distinguish true spots from artifacts, the quality and intensity of the training vary among laboratories, and even technicians working in the same laboratory often undergo different training, contributing to the systematic variation of spot counts between technicians. One Taiwanese laboratory determines test results by calculating mean values from spot counts manually read by two technicians to address inter-technician variability [10]. In one Spanish laboratory, test results were determined manually by one technician or, in case of doubt, by two technicians [11]. Franken et al. found that some technicians tended to count more or fewer spots than others, but they did so consistently in all four wells of a given sample [8]. This study showed that the systematic variation in spot counts read by different technicians was nullified by subtracting the spot count in the nil control [8]. Random variation is another source of inter-technician variability and also inherent to any ELISPOT plate imager [12]. Even one spot count difference between different technicians due to random variation could yield different test results, particularly in samples with spot counts around the cutoff.

IGRAs are prone to biological and technical variability, and hence, the borderline category has been recommended to define genuine conversions and reversions [13]. Our study showed that the borderline category had the lowest concordance (56.76%), which was consistent with the previous study [7]. Such low concordance is probably attributable to the narrow range (5 to 7 spots) of the FDA-recommended borderline category. Higher concordance is expected in laboratories adopting the borderline category with the wider range (4 to 8 spots) suggested by van Zyl-Smit et al. [14]. Due to high variability, we recommend that borderline test results by an ELISPOT plate imager should be manually confirmed. Most borderline results resolve into positive or negative test results on retesting [15, 16], but manual confirmation of borderline test results could be more cost-effective than retesting by drawing another blood sample. Manual confirmation is expected to determine 8 positive and 33 negative test results from 111 borderline test results read by the ELISPOT plate imager. The workload of manual confirmation varies depending on the proportion of borderline test results read by an ELISPOT plate imager. Although borderline test results read by an ELISPOT plate imager are manually confirmed as positive or negative, clinical decision making should consider the limitations of T-SPOT.*TB* and individual’s risks and benefits of LTBI treatment [17]. Also, laboratories performing the T-SPOT.*TB* should avoid reporting only qualitative test results and provide spot counts to referring clinicians [14].

ELISPOT plate imagers can produce false positive or false negative results, leading to increased healthcare costs despite the presence of borderline category. The possibility of false negative results is considered slim because in this study, most discordant test results resolved to negative test results on retesting. Thus, negative test results by an ELISPOT plate imager can be reported as such without significant risks. Oxford Diagnostic Laboratories manually confirms all positive test results by an ELISPOT plate imager to avoid false positive results [16]. In this study, two positive test results read by the ELISPOT plate imager were potentially false positive because they were classified by manual reading as negative. In those two test results, the higher of net panel A and net panel B spot count was 8 at the positive cutoff. According to Bland-Altman analysis, the agreement of spot counts was good, and variability did not increase with higher spot counts. This finding is consistent with that of Janetzki et al. [18], who demonstrated that the spot count difference between manual reading and an ELISPOT plate imager was the smallest in the range of 0 to 20 spots. Thus, manual confirmation should be confined to positive test results with the maximum net spot count of 8. Before introducing an ELISPOT plate imager, individual laboratories need to understand that different interpretation algorithms generate different test results of T-SPOT.*TB*. Our interpretation algorithm offers a practical guide to them.

ELISPOT plate imagers are programmed to distinguish spots from artifacts and other unwanted signals by using reading parameters such as spot size, intensity of staining, spot gradient, and spot shape [6]. Reading parameters and spot-recognition algorithms differ among ELISPOT plate imagers, thereby leading to disparity in T-SPOT.*TB* test results. Precision of ELISPOT plate imagers for spot counting is good, particularly in a sample well with low spot counts [14]; however, the absence of a gold standard for LTBI testing hampers the assessment of the accuracy of spot counting. ELISPOT plate imagers are fine-tuned to exclude artificial signals created by dead or apoptotic cells and other contaminating cell populations including granulocytes, platelet, or red blood cells [6]. Even with standardized reading parameters, however, perfectly discriminating spots from artifacts is inherently impossible due to the fact that sizes and densities of spots vary depending on quantities and kinetics of cytokines generated by the individual T-cells [19]. Moreover, disruption of spot formation by high granulocyte contamination impedes accurate spot counting [20]. ELISPOT plate imagers can also produce false positive or false negative results in the presence of high background reactivity. Lastly, the accuracy of spot counting is subject to technical factors affecting image acquisition of ELISPOT plate wells such as the quality of digital files, camera settings, and lighting system [6, 21]. Individual laboratories should unequivocally understand sources of undermining the accuracy of spot counting and establish their own standard operating procedure to address them.

## Conclusions

Our study reveals that concordance between manual reading and automated evaluation is high. Our recommended strategy confines manual confirmation to positive test results with the maximum net spot count of 8 and borderline test results due to relatively high discordance. Our recommendations might not be directly applicable to laboratories having an ELISPOT plate imager with different algorithms and reading parameters but could be a practical guide to establishing their own strategies for interpreting the test results read by an ELISPOT plate imager.

## Supporting information

S1 Table Comparison of the qualitative test results read by each technician and ELISPOT plate imager. (DOCX)
