# Supplementary material for: A novel strategy for interpreting the T-SPOT.TB test results read by an ELISPOT plate imager
Source: PLoS One. 2019 Sep 25;14(9):e0222920. doi: 10.1371/journal.pone.0222920 (PMC6760805; doi:10.1371/journal.pone.0222920)
Supplement: S1 Table — (DOCX) [file pone.0222920.s001.docx]

**S1A Table. Comparison of the qualitative test results read by the technician 1 and ELISPOT plate imager.**

|  | ELISPOT plate imager | | | | | |
| --- | --- | --- | --- | --- | --- | --- |
| Technician 1 | Test results | Positive | Borderline | Negative | Invalid | Total |
|  | Positive | 56 | 3 | 0 | 0 | 59 (23.23%) |
|  | Borderline | 0 | 13 | 1 | 0 | 14 (5.51%) |
|  | Negative | 1 | 9 | 164 | 0 | 174 (68.50%) |
|  | Invalid | 0 | 1 | 1 | 5 | 7 (2.76%) |
|  | Total | 57 (22.44%) | 26 (10.24%) | 166 (65.35%) | 5 (1.97%) | 254 (100.00%) |

**S1B Table. Comparison of the qualitative test results read by the technician 2 and ELISPOT plate imager.**

|  | ELISPOT plate imager | | | | | |
| --- | --- | --- | --- | --- | --- | --- |
| Technician 2 | Test results | Positive | Borderline | Negative | Invalid | Total |
|  | Positive | 104 | 0 | 0 | 0 | 104 (26.46%) |
|  | Borderline | 1 | 14 | 1 | 0 | 16 (4.07%) |
|  | Negative | 0 | 11 | 241 | 0 | 252 (64.12%) |
|  | Invalid | 0 | 0 | 1 | 20 | 21 (5.34%) |
|  | Total | 105 (26.72%) | 25 (6.36%) | 243 (61.83%) | 20 (5.09%) | 393 (100.00%) |

**S1C Table. Comparison of the qualitative test results read by the technician 3 and ELISPOT plate imager.**

|  | ELISPOT plate imager | | | | | |
| --- | --- | --- | --- | --- | --- | --- |
| Technician 3 | Test results | Positive | Borderline | Negative | Invalid | Total |
|  | Positive | 179 | 4 | 0 | 0 | 183 (26.48%) |
|  | Borderline | 3 | 33 | 2 | 0 | 38 (5.50%) |
|  | Negative | 1 | 13 | 434 | 0 | 448 (64.83%) |
|  | Invalid | 0 | 6 | 2 | 14 | 22 (3.18%) |
|  | Total | 183 (26.48%) | 56 (8.10%) | 438 (63.39%) | 14 (2.03%) | 691 (100.00%) |

**S1D Table. Comparison of the qualitative test results read by the technician 4 and ELISPOT plate imager.**

|  | ELISPOT plate imager | | | | | |
| --- | --- | --- | --- | --- | --- | --- |
| Technician 4 | Test results | Positive | Borderline | Negative | Invalid | Total |
|  | Positive | 20 | 1 | 0 | 0 | 21 (24.71%) |
|  | Borderline | 1 | 3 | 1 | 0 | 5 (5.88%) |
|  | Negative | 0 | 0 | 54 | 0 | 54 (63.53%) |
|  | Invalid | 1 | 0 | 0 | 4 | 5 (5.88%) |
|  | Total | 22 (25.88%) | 4 (4.71%) | 55 (64.71%) | 4 (4.71%) | 85 (100.00%) |
